# Four‐dimensional treatment planning strategies for dynamic tumor tracking

**DOI:** 10.1002/acm2.14269

**Published:** 2024-01-18

**Authors:** Emilie E. Carpentier, Ronan L. McDermott, Marie‐Laure A. Camborde, Tania Karan, Alanah M. Bergman, Ante Mestrovic

**Affiliations:** ^1^ Department of Physics and Astronomy University of British Columbia Vancouver British Columbia Canada; ^2^ Department of Medical Physics BC Cancer – Vancouver Vancouver British Columbia Canada; ^3^ Radiation Oncology BC Cancer Vancouver Vancouver British Columbia Canada

**Keywords:** 4D dose calculation, 4D treatment planning, dynamic tumor tracking, gimballed linac system

## Abstract

**Introduction:**

Dynamic tumor tracking (DTT) is a motion management technique where the radiation beam follows a moving tumor in real time. Not modelling DTT beam motion in the treatment planning system leaves an organ at risk (OAR) vulnerable to exceeding its dose limit. This work investigates two planning strategies for DTT plans, the “Boolean OAR Method” and the “Aperture Sorting Method,” to determine if they can successfully spare an OAR while maintaining sufficient target coverage.

**Materials and methods:**

A step‐and‐shoot intensity modulated radiation therapy (sIMRT) treatment plan was re‐optimized for 10 previously treated liver stereotactic ablative radiotherapy patients who each had one OAR very close to the target. Two planning strategies were investigated to determine which is more effective at sparing an OAR while maintaining target coverage: (1) the “Boolean OAR Method” created a union of an OAR's contours from two breathing phases (exhale and inhale) on the exhale phase (the planning CT) and protected this combined OAR during plan optimization, (2) the “Aperture Sorting Method” assigned apertures to the breathing phase where they contributed the least to an OAR's maximum dose.

**Results:**

All 10 OARs exceeded their dose constraints on the original plan four‐dimensional (4D) dose distributions and average target coverage was V_100%_ = 91.3% ± 2.9% (ranging from 85.1% to 94.8%). The “Boolean OAR Method” spared 7/10 OARs, and mean target coverage decreased to V_100%_ = 87.1% ± 3.8% (ranging from 80.7% to 93.7%). The “Aperture Sorting Method” spared 9/10 OARs and the mean target coverage remained high at V_100%_ = 91.7% ± 2.8% (ranging from 84.9% to 94.5%).

**Conclusions:**

4D planning strategies are simple to implement and can improve OAR sparing during DTT treatments. The “Boolean OAR Method” improved sparing of OARs but target coverage was reduced. The “Aperture Sorting Method” further improved sparing of OARs and maintained target coverage.

## 1 INTRODUCTION

Patient motion during radiation therapy treatments is a well‐known concern that can lead to tumor under‐dosage and healthy tissue over‐dosage.^1^ Intrafractional respiratory motion in particular requires special consideration when treating abdominal and thoracic tumors.^2^, ^3^ Many techniques have been developed to address respiratory motion during radiation treatments to ensure the tumor receives the prescribed dose, such as motion‐encompassing margins around the target,^4^ respiratory gating,^5^ and real‐time tumor tracking.^6^, ^7^, ^8^, ^9^

The Vero4DRT (Vero) is a real‐time tumor tracking system with a gimbal‐mounted linear accelerator (linac) head.^9^ The gimbal system can pan and tilt the radiation beam to irradiate the tumor anywhere within a ±4.2 cm plane at isocenter.^10^ The Vero has an O‐ring gantry that can rotate around the patient's superior‐inferior and anterior‐posterior axes, allowing non‐coplanar beam deliveries. The system is equipped with two sets of orthogonal kV imagers and detectors and a couch with five degrees of freedom (ring rotation offering a sixth degree of freedom).^9^ Prior to a DTT treatment, the Vero builds a four‐dimensional (4D) correlation model between the motion of gold fiducial markers (fids) implanted in the patient near their target and infrared (IR) markers placed on the patient's chest. The fids’ motion is monitored by the orthogonal kV imagers and the IR marker motion is recorded using an IR camera in the treatment room. During treatment, the real‐time motion of the IR markers on the patient's chest is input into the 4D correlation model to predict the position of the fiducial markers, and therefore the target, in real‐time. This information guides the gimbal system on how to pan/tilt the beam.^6^, ^11^, ^12^ The tracking error between the position of the fiducial markers and the gimbals is less than 3.08 mm on average.^13^

Currently, DTT plans at the authors’ center are created, optimized and evaluated on a single computed tomography (CT) image, despite treatment occurring over the entire breathing cycle. Additionally, there are no commercially available treatment planning systems (TPS) that model the Vero's panning and tilting beam motion. Neglecting to model the beam's panning/tilting geometry and the respiratory motion of the patient's anatomy can produce a dose distribution that is inaccurate. While target under‐dosage is less of a concern for DTT treatments since the beam is following and irradiating the tumor, an OAR may still be vulnerable to exceeding its dose limit during other breathing phases, as shown by Carpentier et al.^14^ Performing a 4D dose calculation of a treatment plan would provide more accurate information about an OAR's maximum dose during treatment.

A 4D dose calculation requires modelling the beam's tracking motion while re‐calculating the plan on multiple breathing phases, subsequently accumulating these dose distributions on a reference phase CT image. A 4D dose calculation can be conducted in the TPS^14^ or with Monte Carlo (MC)^15^ using 2 or 10 breathing phases from a patient's 4DCT image set. Using only 2 breathing phases, the inhale (0%) and exhale (50%) phases, with patient‐specific weightings gives similar results as using all 10 breathing phases but requires less time and resources.^14^ A 10‐phase 4D dose calculation of a treatment plan can be complete in under an hour, and a 2‐phase 4D dose calculation can be complete in about 10 min. Accumulating dose distributions from different breathing phases into a single 4D dose distribution provides clinically useful information about the dose to OARs during the DTT treatment.

However, a challenge associated with the previously mentioned work is when a 4D dose calculation indicates an OAR will exceed its dose limit. In this case, the plan needs to be re‐optimized on the planning CT image and a 4D dose distribution needs to be re‐calculated. This process would repeat until the 4D dose calculation indicates until the OAR is below its dose limit. This is inefficient and time consuming as it may require several iterations. A more effective planning procedure would include treatment planning strategies that can be implemented during plan creation/optimization on the planning CT image to ensure an OAR will be below its dose limit after a 4D dose calculation. This can be accomplished by incorporating information from multiple breathing phases into the plan development process.

This work explores the efficacy of two planning strategies that can be employed to improve safety and efficiency when creating a DTT treatment plan. To the author's knowledge, such planning strategies have not been reported previously. Both planning strategies were designed to spare an OAR over the entire breathing cycle while optimizing the plan on a single breathing phase only. They are simple to implement in the TPS and could be easily adapted for any real‐time tumor tracking system. Adopting planning strategies into the treatment planning protocol may improve the plan's quality and reduce the time required to produce an acceptable plan.

## 2 MATERIALS AND METHODS

The original DTT treatment plan was optimized on the exhale phase CT following the current clinical workflow at the authors’ center. Both planning strategies were then applied to this original plan to evaluate their effectiveness. The evaluation was based on dosimetric results from a 4D dose calculation of the original plan when no planning strategies were used compared to when each strategy was applied individually.

### 2.1 Original DTT plan

A 7‐beam step‐and‐shoot intensity modulated radiation therapy (sIMRT) treatment plan was optimized in the TPS (RayStation, RaySearch Laboratories) on a single breathing phase image (the exhale phase of a 4DCT). The exhale phase was chosen for planning because individuals naturally tend to pause when exhaling, thereby spending most of the respiratory cycle (>50% of the time) in exhalation. Therefore, this plan would be created on an image that represents the patient's anatomy during a majority of their breathing. All dose constraints were met and target coverage was maximized, with the goal for target coverage being V_100%_ = 95% to the planning target volume (PTV). The PTV was a 5 mm margin added to the clinical target volume (CTV).

### 2.2 4D dose calculations

4D dose distributions calculate the dose on multiple breathing phases while modelling the appropriate panning and tilting geometry of the beam for each phase.^14^ In this study two breathing phases were used: the plan optimized on the exhale phase (i.e., the original DTT plan) was transferred to the inhale phase and the beams’ angles were altered to model the appropriate panning/tilting that would occur during inhalation. The inhale dose distribution was re‐calculated and deformed back to the exhale phase, where it was then accumulated with the exhale phase dose distribution using patient‐specific phase weightings. These weightings were determined from the patient's breathing trace, acquired at the time of their 4DCT scan. The breathing trace's peak‐to‐peak amplitude was divided into inhalation and exhalation, and the percent of time spent in each half of the breathing cycle, averaged over all cycles available in the breathing trace, was used to determine the weightings for the inhale and exhale phases. This method for a 2‐breathing‐phase 4D dose calculation that models panning/tilting, and how the weightings are determined, is outlined in further detail in Carpentier et al.^14^

### 2.3 Planning strategy #1: Boolean OAR method

During a DTT treatment, the beam follows the implanted fiducial markers which are a surrogate for the target (PTV). However, the relative position of the OARs with respect to the target might change over the breathing cycle. Therefore, the position of the OARs may be “blurred” with respect to the target position (and the beam). To account for this blurring of the OARs’ position, the “Boolean OAR method” was developed.

**FIGURE 1:**
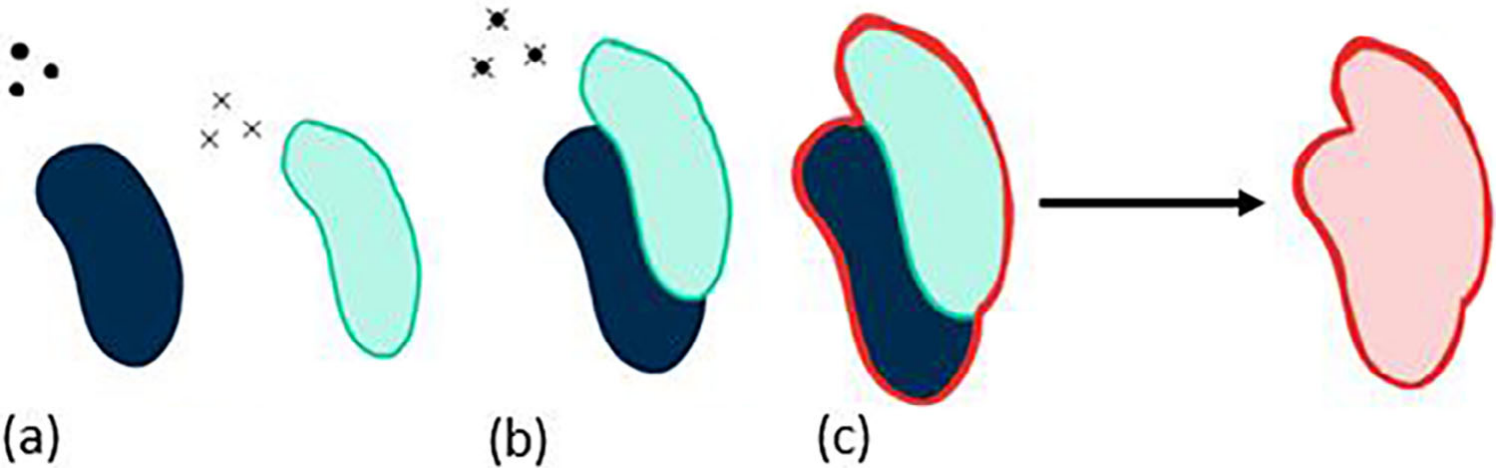
Creating the boolean OAR. (a) The exhale phase OAR (dark blue) and inhale phase OAR (light green) with fids indicated by dots (exhale) and x's (inhale). (b) The inhale and exhale phases are registered based on the position of fiducial markers, and the inhale OAR is then transferred to the exhale phase. (c) The union of the contours becomes the boolean OAR that is protected in the subsequent plan optimization.

The “Boolean OAR” method is similar to the planning organ at risk volume (PRV) technique. A PRV is a margin added to an OAR on a CT image that represents population‐based systematic and random errors in the position of the OAR.^16^ However, the “Boolean OAR” method does not add a margin to the OAR of concern. It instead combines the OAR contour from both the exhale and inhale CT images of the patient's 4DCT. First, the images were registered by aligning the fiducial markers implanted near the target on each phase. This registration aligned the patient's images in a way that reflected the beam's eye view of the anatomy since the Vero tracks the fiducial markers as a surrogate for tumor motion. Then, the contour on the inhale image was transferred to the exhale image based on this registration. Next, a boolean union of the two contours was created on the exhale phase, providing a contour of the OAR that encompasses its patient‐specific position during the extreme ends of the breathing cycle (inhalation and exhalation) according to the beam's eye view. The boolean contour creation steps are outlined in Figure 1.

If a 4D dose calculation of the original DTT plan identified that an OAR crossed its maximum dose limit, the “Boolean OAR” method was employed to try to reduce the OAR's maximum dose. The plan was still re‐optimized on the exhale phase, but now the boolean OAR contour was protected instead of just the exhale phase contour in the optimization functions. This new plan then underwent a 4D dose calculation to determine if using the boolean OAR in the optimization functions was sufficient to protect it.

### 2.4 Planning strategy #2: Aperture sorting method

As with the “Boolean OAR” method, if a 4D dose calculation of the original DTT plan identified that an OAR crossed its maximum dose limit, the “Aperture Sorting” method was also used to attempt to reduce the OAR's maximum dose. The “Aperture Sorting” method would consider the contribution each aperture makes to an OAR's maximum dose on either breathing phase (exhale or inhale) and assign certain apertures to be delivered only during the phase where they contribute the least dose. For example, if an aperture contributed more to an OARs’ maximum dose during inhalation than exhalation, that aperture would be designated to be delivered only when the patient is exhaling. Not every aperture needed to be sorted; this method prioritized assigning apertures that had the greatest dosimetric difference on one phase over the other and stopped sorting apertures once an OAR's maximum dose went below its dose limit. Unlike the “Boolean OAR” method, “Aperture Sorting” did not require re‐optimizing the original plan and used the apertures that were created during the optimization of the original plan.

**FIGURE 2:**
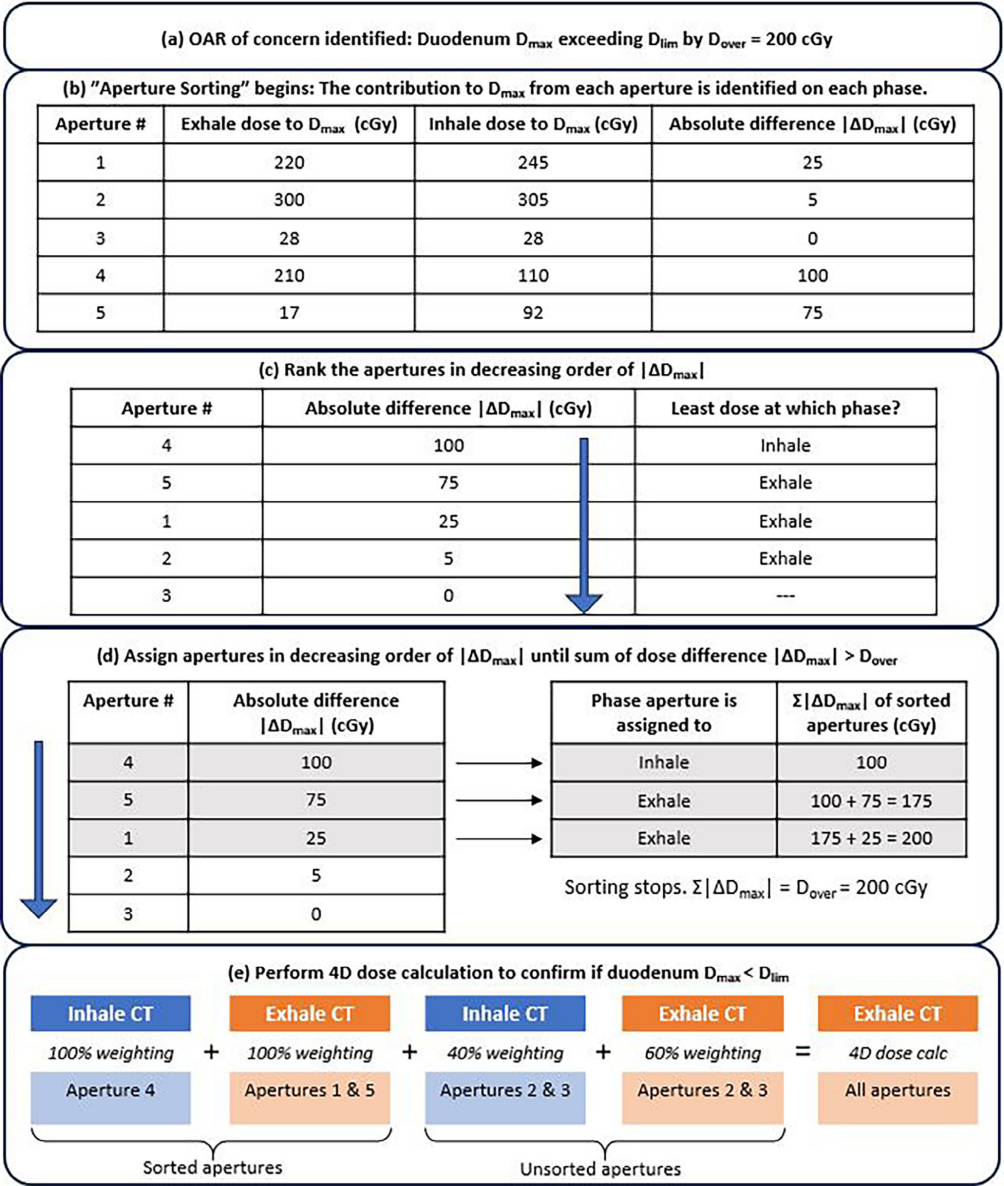
A simplified example of how the “Aperture Sorting” method works. This example uses only five apertures to describe the “Aperture Sorting” script. (a) A 4D dose calculation of the original DTT plan has identified that the patient's duodenum would exceed its dose limit by 200 cGy. (b) The dose contribution each aperture makes to the maximum dose on the duodenum is determined for both the exhale and inhale phase images. The absolute difference in dose between both phases, |ΔD_max_|, is also recorded. (c) Apertures that have a large difference in contribution to the maximum dose when delivered on one phase over the other are ranked higher priority to sort. (d) Starting at the top of the list in c), apertures are assigned to the phase where they contribute the least to the duodenum's maximum dose. The script keeps track of the impact these assignments should have on the duodenum's maximum dose, and stops sorting when the dose difference sums to D_over_ = 200 cGy. (e) A 4D dose calculation is performed to confirm the new maximum dose to the duodenum.

The “Aperture Sorting” method is fully automated and implemented into the TPS via a script. Figure 2 shows a simplified example using only five apertures to demonstrate how this script implements the “Aperture Sorting” method on an existing plan. The script first identified how much the OAR maximum dose, D_max_, needed to decrease by to meet its maximum dose constraint, D_lim_ (Figure 2, step a). Next, each aperture's contribution to the OAR's maximum dose was calculated on either breathing phase. Apertures that contributed more to the maximum dose on one phase over the other were identified and the absolute difference in maximum dose contribution (|ΔD_max_|) was recorded (Figure 2, step b). In decreasing order, starting with apertures that had the greatest dosimetric difference on one phase over the other, (Figure 2, step c), these apertures were “sorted” and assigned to be delivered only during the breathing phase where they contributed the least to the maximum dose (Figure 2, step d). When the sum of |ΔD_max_| of all apertures sorted thus far was equal to or greater than D_max_—D_lim_ = D_over_ (the targeted maximum dose reduction), a 4D dose calculation was performed to re‐calculate D_max_ to the OAR and confirm if enough apertures had been assigned to certain phases to spare the organ. To perform a 4D dose calculation with some sorted apertures, the unsorted apertures were still calculated on both breathing phases with patient specific weightings as usual, and the sorted apertures were calculated only on the phase they were assigned to. All these dose distributions (unsorted apertures on both phases, appropriately weighted, and sorted apertures on their respective phases with 100% weightings) were accumulated on the exhale phase into one 4D dose distribution (Figure 2, step e). If D_max_ was still greater than D_lim_, the “Aperture Sorting” method continued to sort through the list of remaining unsorted apertures (returning to step d in Figure 2 and sorting the remaining apertures, shown by unshaded cells). Alternatively, if D_max_ was now below D_lim_, the script stopped. All apertures assigned to the exhale phase, inhale phase, and all apertures that remained unsorted, were saved to separate plans.

**FIGURE 3:**
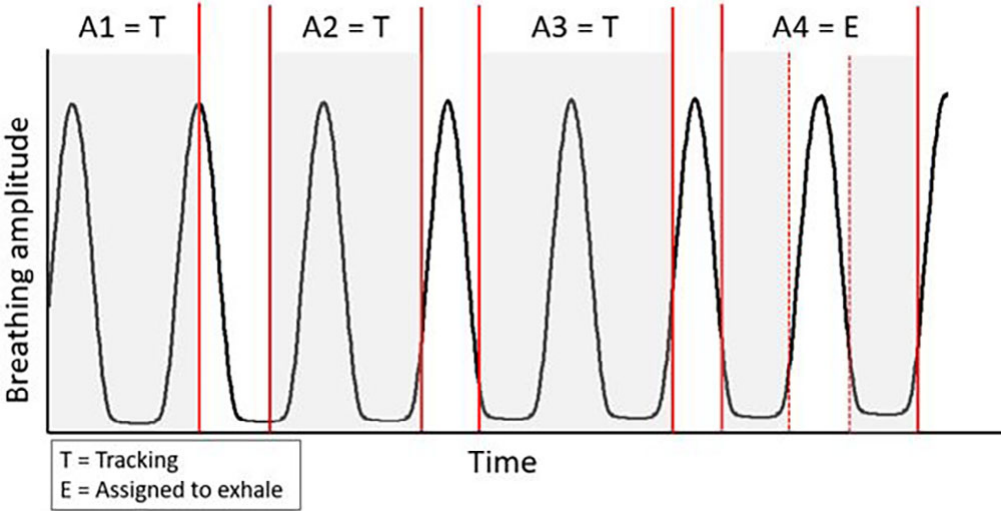
An example of delivering a plan after using “Aperture Sorting”. A simplified example of delivering a sIMRT plan after the “Aperture Sorting” method was used. In this example, apertures 1−4 (A1‐A4) belong to beam #1 and are delivered consecutively. Only A4 was assigned to the exhale phase, and A1‐A3 can be delivered on any breathing phase with regular DTT. The shaded regions represent when the beam is on; it turns off between each aperture as the multi‐leaf collimators move to the new position of the next aperture. A1‐A3 can be delivered during any breathing phase with DTT, whereas the beam can only be turned on to deliver A4 when the patient is in the exhale phase.

To deliver a plan that used the “Aperture Sorting” method, sorted apertures would need to be delivered during the phase they were assigned to. Therefore, these treatments with sorted apertures would have a “gating‐like” element for the sorted apertures, and the apertures that did not need to be sorted can be delivered with DTT regularly. An example of such a delivery is shown in Figure 3. This special requirement for treatment delivery is likely possible with the existing technology at the treatment unit. For example, the Vero's Exactrac system outputs a plot on the computer in the treatment console room indicating the real‐time position of the IR markers on the patient's chest as they breathe during treatment. This can inform the system when the patient is in a specific breathing phase and the beam can be turned on to deliver apertures assigned to that phase and then turned off when the patient is moving out of that breathing phase. Manually switching the beam on and off can be combined with coaching the patient to hold their breath in a specific phase using the microphone and speaker system between the console area and the treatment room. This coaching technique while manually turning the beam on and off is already used at the author's center for left breast radiation therapy using deep‐inspiration breath hold. This is one example of how to deliver a plan using the “Aperture Sorting” method, although other solutions may also be viable.

### 2.5 Phantom case

**FIGURE 4:**
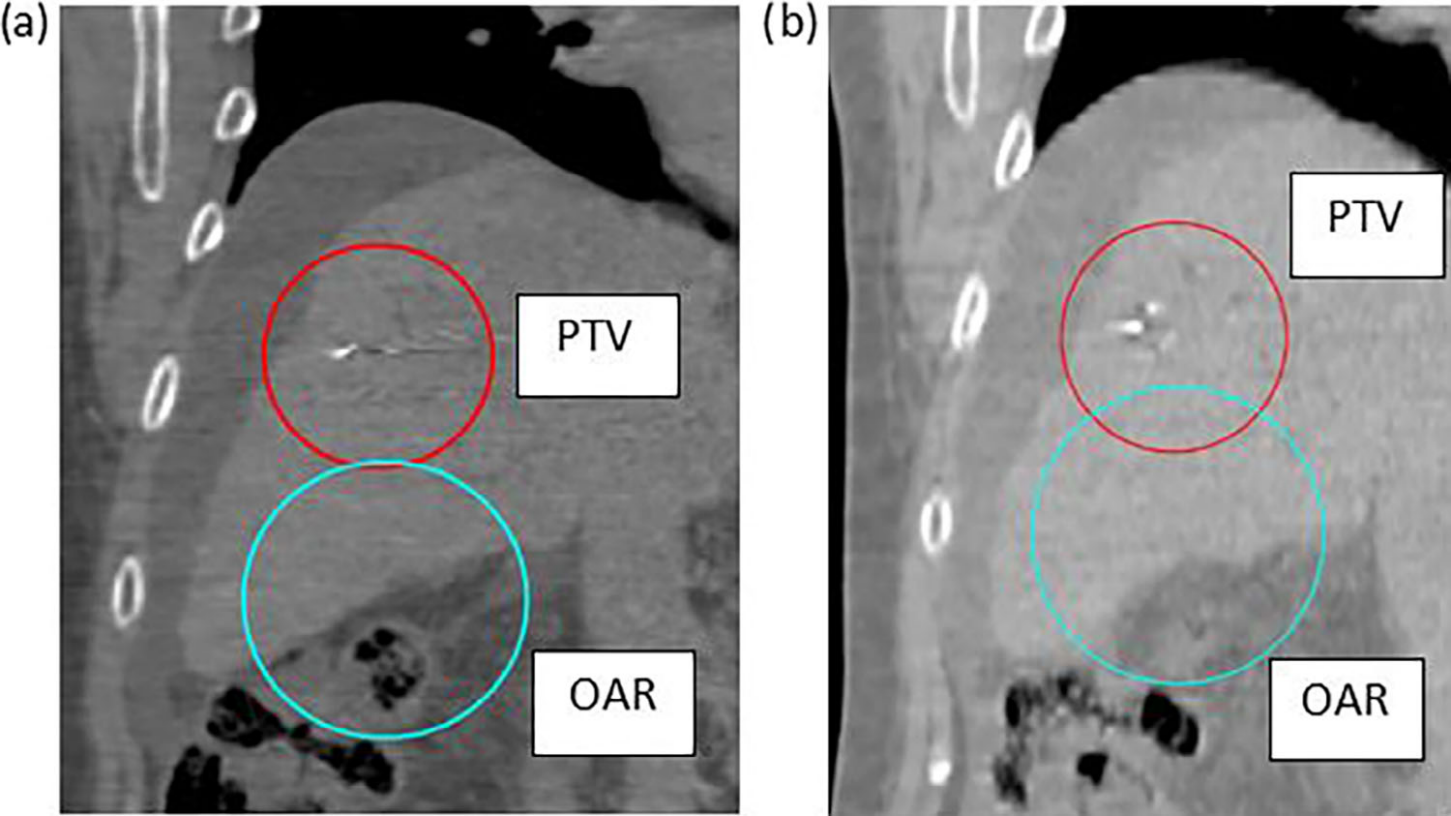
Images of the phantom case setup. Coronal CT images from the phantom dataset with the body contour set to water density. The exhale (a) and inhale (b) CT images are shown with the OAR (light blue) and PTV (red) contours. Note: the actual Hounsfield units are displayed in the image, however they were set to 0 for all voxels.

A simple phantom case was created to test these strategies initially. The body contour of a patient's CT image was set to water density to be used as a test case. An artificial spherical “target” was created in the liver on the exhale phase image and a spherical “OAR” was placed inferior to it, given a dose constraint of 2800 cGy that is representative of a typical stereotactic ablative radiotherapy (SABR) dose constraint. Similarly, on the inhale phase the “target” was placed in the liver and the “OAR” was placed inferiorly, now in closer proximity to the target. This phantom models common respiratory motion where a target in the liver moves closer to inferior OARs when a patient inhales. The phantom provided a simplified example with which to test these planning strategies that eliminated any effects due to tissue heterogeneities and provided the opportunity to control where the “target” and “OAR” are placed. The phantom anatomy created for this study is shown in Figure 4.

A 7‐beam sIMRT plan with a prescription of 45 Gy in 5 fractions was optimized for this phantom on the exhale phase (original DTT plan). All dose constraints were met by this plan and the target's coverage was maximized. A 4D dose calculation of this plan was conducted, and then both planning strategies were applied to the original plan to demonstrate their effectiveness.

### 2.6 Clinical study

The CT data of 10 previous liver SABR patients was used in this study. These patients were selected because they each had one concerning OAR in very close proximity to the PTV, as well as implanted fiducial markers near the target. The OARs of concern included the large bowel, duodenum, stomach, heart, and major vessels. The original DTT plan was optimized for each dataset on the exhale phase CT image so that all dose constraints were met and target coverage was maximized. Treatment prescriptions used in this study were 45 Gy in 5 fractions, 45 Gy in 3 fractions, 54 Gy in 3 fractions, and 54 Gy in 5 fractions. The patients’ breathing traces had inhale/exhale weightings ranging from 39%/61% to 26%/74%. 4D dose calculations of each patient's original plan were performed and both planning strategies were applied to determine how well they perform in real clinical cases.

## 3 RESULTS

### 3.1 Phantom case

#### 3.1.1 Original DTT plan

The original plan optimized on the exhale image met all dose constraints and target coverage was maximized (V_100%_ = 94.4%). The OAR of concern directly inferior to the target had a dose limit of 2800 cGy and the maximum dose of the original plan on the exhale phase was 2762 cGy. A 4D dose calculation of the original plan (using an exhale/inhale weighting of 60%/40%) indicated the OAR inferior to the target was exceeding its dose constraint; the OAR's maximum dose was now 3881 cGy and target coverage was maintained at V_100%_ = 94.4%.

#### 3.1.2 Planning strategies: Boolean OAR and aperture sorting

After applying the “Boolean OAR” method to the original plan and re‐calculating the 4D dose distribution, the maximum dose to the OAR was reduced to D_max_ = 2038 cGy but target coverage dropped to V_100%_ = 78.9%. Applying the “Aperture Sorting” method to the original plan and re‐calculating the 4D dose distribution resulted in a maximum dose of D_max_ = 2791 cGy to the OAR of concern and target coverage remained similar to what was originally achieved: V_100%_ = 93.4%. Out of 28 total apertures in the 7‐beam plan, 15 needed sorting and were assigned to the exhale phase. The dosimetric results for the phantom case are summarized in Table 1.

**TABLE 1:** Dosimetric results for phantom case, with and without planning strategies.

	3D dose calculation (Original plan)	4D dose calculation (Original plan)	4D dose calculation (Boolean OAR method)	4D dose calculation (Aperture sorting method)
**OAR D_max_ (cGy)**	2762	3881	2038	2791
**PTV V_100%_ (%)**	94.4	94.4	78.9	93.4

*Note*: The maximum dose (D_max_, cGy) to the artificial spherical OAR inferior to the target, and the PTV coverage (V_100%_, %), for the phantom case is tabulated for each method used to calculate the dose distribution. “3D dose calculation” refers to the original DTT plan calculated on the exhale phase, and “4D dose calculation” refers to the original DTT plan calculated on the weighted exhale and inhale phases.

### 3.2 Clinical study

#### 3.2.1 Original DTT plan

For all 10 clinical datasets used in this study, their original plan optimized on the exhale phase met all dose constraints and the mean target coverage was V_100%_ = 93.1% (range = [86.2%, 95.9%]). After a 4D dose calculation of these plans, each patient had one OAR exceed its dose limit by 1.1% to 38% (50 cGy to 1100 cGy above the dose limit). Target coverage remained similar to the original coverage (mean V_100%_ = 91.3% ± 2.9%, range = [85.1%, 94.8%]).

#### 3.2.2 Planning strategies: Boolean OAR and aperture sorting

**FIGURE 5:**
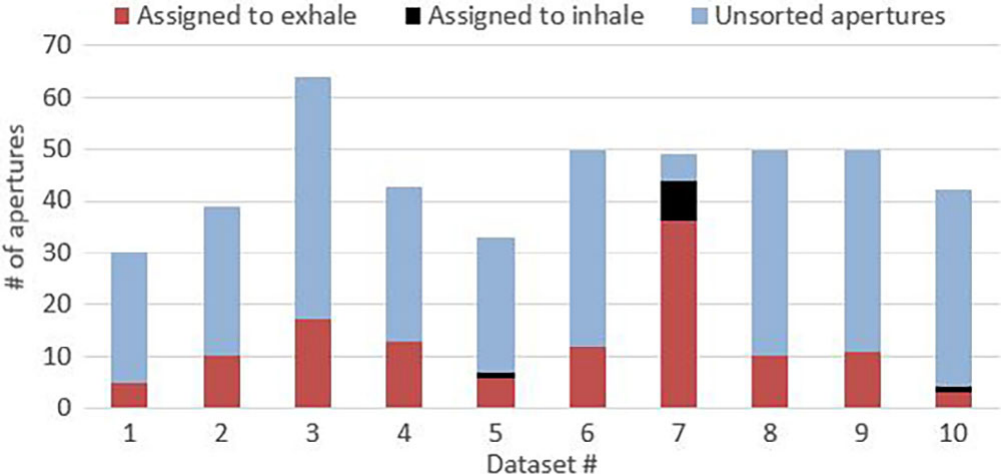
A plot of the number of sorted apertures. The number of sorted apertures and the number of unsorted apertures for each clinical dataset after the “Aperture Sorting” method was used. It was unsuccessful at bringing D_max_ below the assigned dose limit for dataset #7 and attempted to sort every aperture; the five unsorted apertures contributed equally to both breathing phases.

The “Boolean OAR” method successfully spared 7 of the 10 OARs that had been exceeding their dose limit. In some cases, sparing was more than necessary and the maximum dose was reduced up to 500 cGy below the allowed dose limit. As a result, target coverage was much lower: V_100%_ = 87.1% ± 3.8% on average (range = [80.7%, 93.7%]). The “Aperture Sorting” method successfully spared 9 of the 10 OARs; the one that was not spared had its maximum dose reduced by 564 cGy and was only 5 cGy over its limit after the planning strategy had been used. The “Aperture Sorting” method maintained target coverage similar to what the original plans achieved after a 4D dose calculation; the mean coverage was V_100%_ = 91.7% ± 2.8% (range = [84.9%, 94.5%]). The fraction of apertures that needed sorting to successfully bring the maximum dose below its dose limit are shown in Figure 5 and ranged from 4/42 to 13/43 (9.5%–30.2% of the total apertures in the plan). These results from the clinical datasets are summarized in Table 2.

**TABLE 2:** Dosimetric results for clinical datasets, with and without planning strategies.

			*3D dose calculation* (Original plan)		*4D dose calculation* (Original plan)		*4D dose calculation* (Boolean OAR method)		*4D dose calculation* (Aperture sorting method)	
Dataset #	OAR of concern	Dose limit (cGy)	OAR D_max_ (cGy)	PTV V_100%_ (%)	OAR D_max_ (cGy)	PTV V_100%_ (%)	OAR D_max_ (cGy)	PTV V_100%_ (%)	OAR D_max_ (cGy)	PTV V_100%_ (%)
1	Duodenum	3200	3152	94.21%	3442	94.68%	3327	89.16%	3197	94.42%
2	Duodenum	2220	2182	94.63%	2821	93.11%	2016	82.73%	2209	93.37%
3	Large bowel	2820	2798	94.59%	3080	89.30%	2423	87%	2817	92.52%
4	Large bowel	3800	3773	94.87%	4049	94.76%	3873	93.73%	3786	94.51%
5	Duodenum	3200	3185	86.23%	3363	85.12%	3163	84.32%	3195	84.93%
6	Duodenum	2220	2192	95.89%	2506	93.15%	2294	91.70%	2201	93.89%
7	Heart	3800	3744	92.58%	4369	92.69%	3291	80.65%	3805	92.29%
8	Great vessels	4400	4358	90.63%	4539	88.41%	4368	87.04%	4398	88.68%
9	Stomach	2220	2174	92.37%	2380	90.64%	2098	85.80%	2217	91.44%
10	Great vessels	4500	4473	94.96%	4548	91.35%	4416	89.07%	4459	90.50%

*Note*: The maximum dose (D_max_, cGy) and the PTV coverage (V_100%_, %) for the 10 clinical datasets is tabulated for the original DTT plan, after a 4D dose calculation with no planning strategies, and after applying the “Boolean OAR” and “Aperture Sorting” strategies. The dose limit for the same OARs may differ due to a different number of fractions for each dataset. The shaded cells indicate when the maximum dose exceeded the OAR's dose constraint. “3D dose calculation” refers to the original DTT plan calculated on the exhale phase, and “4D dose calculation” refers to the original DTT plan calculated on the weighted exhale and inhale phases.

## 4 DISCUSSION

The planning strategies discussed here were both successful at sparing some OARs. However, there are also challenges associated with each method. The “Boolean OAR” method was fast and simple to implement; the registration of the exhale and inhale images, transferring the inhale contour to the exhale CT, and combining both contours together can be completed in under 5 min using the user interface of the TPS. This makes the “Boolean OAR” method a desirable choice for clinical implementation. However, it does require re‐optimizing the treatment plan that was originally planned on the exhale phase to protect the boolean contour instead. Additionally, because the OAR to protect was much larger than the original OAR on just the exhale phase, target coverage can be lost. Over‐sparing the OAR was also common, reducing the maximum OAR dose unnecessarily low which could sacrifice other aspects of the plan's quality. This method may require several iterations to achieve the desired PTV coverage and OAR sparing. Therefore, for certain patients it may not be an efficient strategy to employ, while for others it may be very effective from its first implementation. For example, Table 2 shows that the “Boolean OAR” method was able to spare the duodenum for dataset #5 and <1% PTV coverage was lost. This is an example of a scenario where the “Boolean OAR” method was an effective choice and only took minutes to implement. The “Boolean OAR” method is similar to the PRV method in that a larger region representing the OAR is protected in the optimization functions. However, the PRV method uses an isotropic margin to encompass positional errors, including respiratory motion. The “Boolean OAR” method uses the exact position of the OAR relative to the beam on each extreme breathing phase to create the expanded contour. This more accurately represents respiratory motion and is patient‐specific.

The “Aperture Sorting” method required initiating a script to run within the TPS. It sorted the necessary apertures created for the original plan on the exhale phase and no further re‐optimization was necessary. This method was also more successful at sparing OARs than the “Boolean OAR” method.  The script was designed to stop sorting apertures once the maximum dose was brought below the OARs’ dose limit so there was no “over‐sparing” that could degrade the plan quality. Target coverage was not sacrificed as with the “Boolean OAR” method and was comparable to the target coverage achieved by the original plan. The time to implement the “Aperture Sorting” method depended on the number of apertures that needed to be sorted and the number of intermediate 4D dose calculations the script performed to check progress. In this study, the scripts took around 1−4 h to complete the “Aperture Sorting” strategy for each of the 10 patients. However, during this time there was no action required by the planner, so it is still considered a low‐maintenance option.

A challenge of the “Aperture Sorting” method is delivering the plan such that the sorted apertures are delivered during the breathing phase they are assigned to. Not all apertures need to be assigned to a certain phase to spare an OAR, and the unsorted apertures can be delivered using DTT normally. However, any sorted apertures would need to be delivered in the breathing phase they are assigned to. The existing technology of the Vero should allow this delivery to be possible, and other linacs that can perform tumor tracking may also already be equipped with the necessary technology. An official clinical protocol would need to be developed to deliver the sorted apertures, and it may add a few extra minutes to the patient's treatment time. Implementing and practicing delivery methods for “Aperture Sorting” plans is an area of future work to determine a specific protocol that would be simple and efficient to adopt.

**FIGURE 6:**
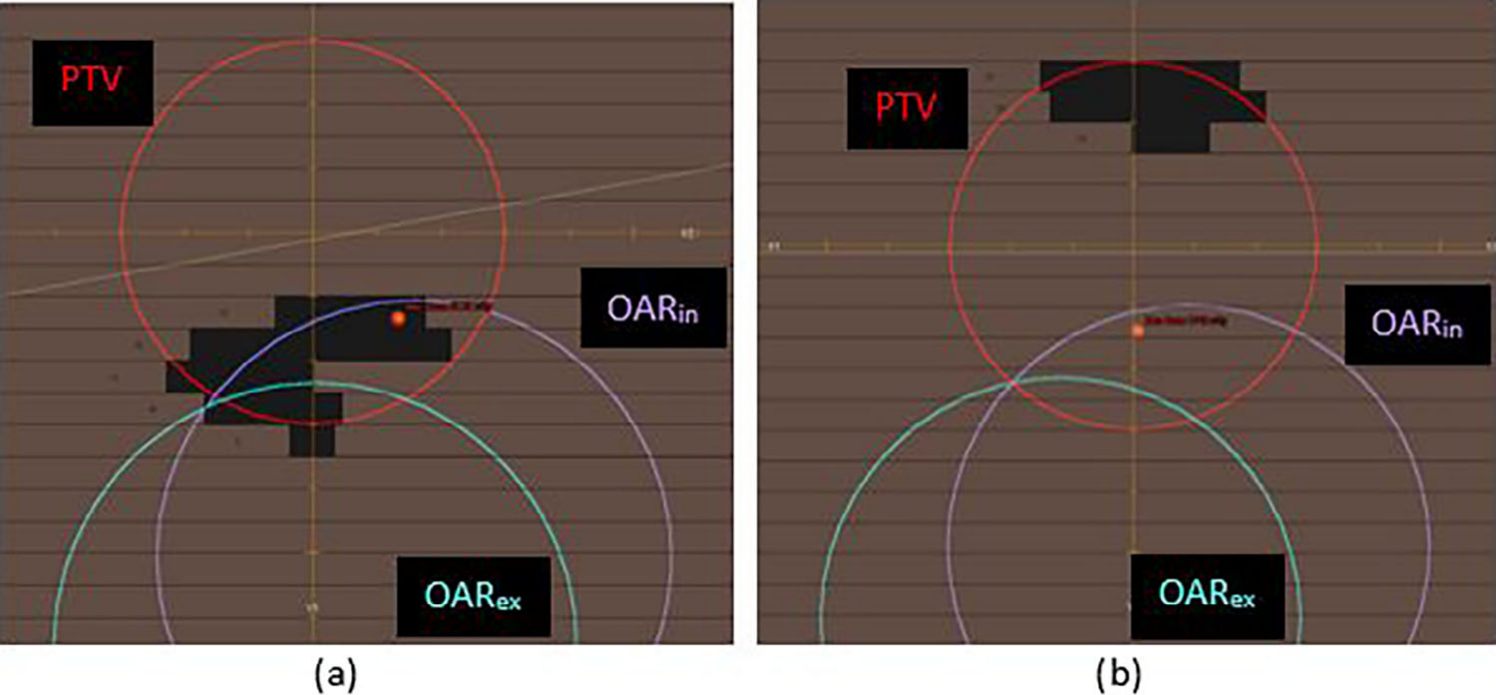
Beam's eye view of different apertures. The beam's eye view of a sorted (a) and unsorted (b) aperture from the water‐density phantom plan. The OAR on the exhale and inhale phases (OAR_ex_ and OAR_in_, respectively) are both shown.

The number of apertures that needed sorting to spare an OAR varied for each plan. Some apertures contributed nearly equal dose during both breathing phases and would have no impact if assigned to one phase only. Other apertures have a large dosimetric difference when delivered on one phase (e.g., inhale) over the other (e.g., exhale), and by assigning a few of these apertures with very large differences the OAR D_max_ will go below D_lim_ and no further sorting is necessary. Figure 6 shows an example of two apertures from the phantom case, one of which was assigned to the exhale phase (Figure 6a) and the other did not need sorting (Figure 6b). The aperture in Figure 6a overlaps with the OAR more on the inhale phase than the exhale phase, highlighting why it has a large dosimetric difference between each phase. However, in Figure 6b the aperture opening is far from the OAR and it contributes a nearly equal low dose during both phases, therefore sorting this aperture would not have much impact. These images are helpful to visualize why some apertures are a greater concern on one breathing phase over another, and why some do not need to be sorted. In total for the phantom case, more than half of the apertures (15 out of 28) needed sorting to the exhale phase. Figure 5 shows the number of apertures sorted to either the exhale or inhale phase for all 10 clinical datasets. Most apertures are sorted to the exhale phase because the plans were optimized for the exhale phase. Dataset #7 was the case where “Aperture Sorting” was unable to reduce the OAR's maximum dose below its dose limit: all apertures were sorted and those remaining that were left unsorted had equal dose contributions to both breathing phases. In general, among all 10 clinical datasets, most apertures were not sorted, so the treatments would still be delivered using DTT primarily.

The planning strategies explored here improve OAR sparing and are efficient; protecting the OAR during the plan creation step and verifying the dose distribution with a 4D dose calculation saves much more time than using no strategies and performing multiple 4D dose calculations after blindly modifying the plan, hoping it improves each time. Assessing the quality of a plan often requires trade‐offs between OAR sparing and PTV coverage, especially in cases like those presented here where the OAR is very close to, if not abutting or overlapping, the PTV. At the authors’ center, a list of priorities ranking which dose constraints are more important than others is created for each patient. Typically, achieving the targeted PTV coverage is a lower priority than OAR maximum dose constraints. If implementing a planning strategy, particularly the “Boolean OAR” method, sacrifices PTV coverage to spare an OAR, the clinician would need to decide if this sacrifice is justified. For example, the “Boolean OAR” method spared dataset #2′s duodenum, but >10% PTV coverage was lost. This would likely be considered unjustifiable, especially if the gross tumor volume coverage is impacted. In contrast, dataset #5′s duodenum was spared using the “Boolean OAR” method and only <1% of the PTV coverage was compromised. This scenario could be considered acceptable.

A limitation of this study is that these planning strategies do not consist of a global 4D optimization using multiple breathing phase CT images to create the plan. These plans are still optimized on the exhale phase only. A more robust 4D plan optimization would likely improve the plan quality and would be an area of future work. Another limitation of these planning strategies is they currently protect only one OAR at a time. A 4D optimization would be able to protect all structures over all breathing phases. However, the procedures and scripts discussed here could theoretically be further developed to include multiple OARs. The quality of the plans produced when multiple OARs are being protected by these strategies has not been investigated, however this could be another area of future work.

Another limitation of 4D treatment planning strategies and 4D plan optimizations is the assumption that the correlation between external motion (e.g., IR markers) and the relative positions of the patient's internal anatomy stays consistent between 4DCT imaging and treatment. This is a challenge for many motion management solutions, and in this case, it could negate the benefits gained from performing a 4D dose calculation and using planning strategies. Since the Vero tracks the motion of external markers to predict the location of internal fiducial markers, a surrogate for the tumor, 4D dose calculations also use fiducial marker displacement on the CT images to accumulate dose from multiple breathing phases. Differences in the correlation of the position of the fiducial markers relative to OARs between the images used for planning and during treatment can impact target and OAR dose, potentially rendering the optimized plan unsafe for healthy organs near the target. Therefore, if 4D planning strategies and 4D dose calculations are used to develop a treatment plan, it is important that the correlation between external motion (e.g., IR markers) and the patient's internal anatomy (relative positions of the PTV and OARs) stays consistent between 4DCT imaging and treatment.

Previous studies have also explored the importance and necessity of 4D dose calculations and plan optimizations. Chan et al. (2013) compared 3D and 4D VMAT dose calculations as well as 4D plan optimizations and dose calculations on the Cyberknife system.^17^ For an OAR that was in extremely close proximity to the PTV, the VMAT 4D dose calculation predicted it would exceed its dose limit when the 3D dose calculation did not. This agrees with previous findings that OARs in close proximity to the PTV may exceed their dose constraint from a 4D dose calculation, despite a 3D dose calculation indicating the OAR is safe, particularly if significant beam motion occurs such as during DTT.^14^ However, in the work presented by Chan et al. the 4D dose calculations for VMAT plans took 3.5 working days to complete and the Cyberknife 4D plan optimizations required two full working days.^17^ Despite the improved dosimetry these techniques provided, it remained unclear if 4D dose calculations were necessary due to the extra time and resources required, and it was recommended that the decision to implement them be patient‐dependent. In the work presented here, 2‐phase 4D dose calculations and planning strategies take less than a few hours to implement. This may offer a clinically feasible solution for those patients with OARs near the target who would greatly benefit from the improved dosimetry of a 4D dose calculation.

## 5 CONCLUSION

The two planning strategies explored in this work have the potential to make DTT plans safer by reducing dose delivered to the OARs over the entire breathing cycle. The “Boolean OAR” method improved sparing of OARs but target coverage was sacrificed. The “Aperture Sorting” method further improved sparing of OARs and maintained acceptable target coverage. Both planning strategies could be adapted for any tracking system to improve planning efficiency and safety of DTT plans.

## AUTHOR CONTRIBUTIONS

All authors on this manuscript contributed to the conception of this work or the acquisition, analysis, and interpretation of the data. Everyone was either involved in drafting the work or made critical revisions prior to submission. All authors approved this final version being submitted and agree to be accountable to the work presented.

## CONFLICT OF INTEREST STATEMENTS

The authors declare no conflicts of interest.

## Data Availability

The data that support the findings of this study are available on request from the corresponding author. The data are not publicly available due to privacy or ethical restrictions.
